# One Third of Malignant Pleural Mesothelioma Shows High Immunohistochemical Expression of MSLN or CXCR4 Which Indicates Potent Candidates for Endo-Radiotherapy

**DOI:** 10.3390/ijms24076356

**Published:** 2023-03-28

**Authors:** Thomas Hager, Sabrina Borchert, Michael Wessolly, Alexander Mathilakathu, Elena Mairinger, Jens Kollmeier, Thomas Mairinger, Balazs Hegedus, Kristina Greimelmaier, Jeremias Wohlschlaeger, Ken Herrmann, Fabian Dominik Mairinger

**Affiliations:** 1 Institute of Pathology, University Hospital Essen, University of Duisburg-Essen, 45147 Essen, Germany; 2 Department of Pathology, Diakonissenkrankenhaus Flensburg, 24939 Flensburg, Germany; 3 Department of Pneumology, Helios Klinikum Emil von Behring, 14165 Berlin, Germany; 4 Department of Pathology, Helios Klinikum Emil von Behring, 14165 Berlin, Germany; 5 Ruhrlandklinik, West German Lung Centre, University Hospital Essen, University of Duisburg-Essen, 45239 Essen, Germany; 6 Department of Thoracic Surgery and Thoracical Endoscopy, Ruhrlandklinik, University Hospital Essen, University of Duisburg-Essen, 45239 Essen, Germany; 7 Clinic for Nuclear Medicine, University Hospital Essen, University of Duisburg-Essen, 45147 Essen, Germany

**Keywords:** pleural mesothelioma, biomarkers, CXCR4, MSLN, radioligand, endo-radiotherapy, theranostics, methylation

## Abstract

Malignant pleural mesothelioma (MPM) is a mainly asbestos-related tumour associated with a very poor prognosis. Therapeutic approaches include multimodal therapy and chemotherapeutics, with cisplatin being the drug of choice, but response rates of only up to 14% indicate very poor outcomes. Effective treatment options are lacking. Besides the diagnostic usage of radioligands in positron emission tomography (PET)/computed tomography (CT), the endo-radioligand therapy with Lu177 has been proven as a powerful tool in cancer therapy. Mesothelin (MSLN) and C-XC chemokine receptor 4 (CXCR4) are membrane-bound proteins, expressed in certain cancers, and thus are promising targets for endo-radiotherapy. A significant portion of high MSLN- or CXCR4-expressing tumors within the MPM may open the field for this sophisticated treatment approach in the near future. Formalin-fixed, paraffin-embedded (FFPE) tumour specimens from 105 patients suffering from MPM and treated at the Lung Cancer Centre of Essen and at the Helios Klinikum Emil von Behring Berlin were screened. The tumour samples were arranged in tissue microarrays. We immunohistochemically stained the tumour samples against MSLN and CXCR4. The protein expressions of the stainings were scored by a pathologist by using a semiquantitative method. The data obtained were correlated with the clinical outcome. Overall, 77.1% of the analysed tumours showed CXCR4 protein expression (25.7% of them at high expression level (Score 3)). 48.6% of all samples showed an overall strong staining (Score ≥ 2), 59% of the investigated tumours showed MSLN protein expression (10.5% of them at high expression (Score 3)), and 36.2% of all samples showed an overall strong staining (Score ≥ 2). Our results show significant tissue expression levels, for both CXCR4 and MSLN protein, in a major portion of clinical MPM samples. One-third of patients showed outstanding immunoexpression of at least one of these markers, making them interesting candidates for radioligand-based PET/CT diagnostics and follow-up and furthermore may profit from endo-radiotherapy.

## 1. Introduction

Malignant pleural mesothelioma (MPM) is a biologically aggressive tumour. Which is predominantly related to asbestos exposure and associated with poor prognosis [1,2]. The optimal management of this severe disease is lacking despite practical guidelines that have been proposed [1,2,3,4]. Multimodality therapy, which consists of several cycles of chemotherapy, subsequent surgery and/or radiotherapy, is performed in early stages. Currently, standard chemotherapeutic approaches are the combination of Cis- or Carboplatin with the antifolate pemetrexed [1,3,4,5,6,7,8,9,10,11]. However, treatment with cisplatin shows a response rate of only 14% [9]. Additionally, a median survival of MPM during cisplatin therapy of less than seven months has been reported [9]. Carboplatin treatment showed similar results with response rates that ranged between 6–16% [9,12]. Predictive biomarkers are, however, still lacking [5,6]. In view of the therapeutic limitations, further basic research is needed to provide opportunities for novel treatment strategies [1].

Within the last few years, the usage of radiopharmaceutical ligands in PET/CT such as 68Ga-Prostate-Specific Membrane antigen (PSMA) has already been consolidated in certain cancer entities [13]. Recently, the Food and Drug Administration (FDA) approved 68Ga-PSMA as the first PSMA-targeted PET drug in potentially curable patients with suspected prostate cancer metastasis or for patients with suspected cancer recurrence [14]. Besides its diagnostic usage, the endo-radioligand therapy with Lu177-PSMA has been proven a powerful tool in therapy of advanced prostate cancer [15]. Besides PSMA, there are multiple other targets discussed for their potency in PET/CT diagnostics or radioligand therapy. The fibroblast-activating protein (FAP) is used in 68Ga-Fibroblast activation protein inhibitor (FAPI) PET/CT [16,17] as well as 64Cu- and labelled with 225Ac FAPI combined in a “theranostics” approach [18].

MSLN is a glycosylphosphatidylinositol (GPI)-anchored membrane protein labelled to cell membranes and releasing the soluble megakaryocyte potentiating factor [19]. It is expressed in several solid tumours, including gastric, lung, pancreatic and ovarian cancers, but also in mesothelioma [20]. Besides its potential as an applicant for targeted therapy [19,20], PET imaging using a 64Cu- or 89Zr-labeled monoclonal antibody against MSLN as well as subsequent therapeutical usage of anti-mesothelin antibody-drug conjugate treatment has shown promising results in pancreatic and/or ovarian cancer [21,22]. CXCR4 is a G protein-coupled chemokine receptor belonging to a protein-family which mediates the chemotaxis of cells toward a gradient of chemokines [23]. CXCR4 is essential during embryonic development, affecting cell migration [24,25]. In healthy tissues, it is absent or only lowly expressed [26,27]. Furthermore, it plays an important role in neo-angiogenesis, infection and immunity [24]. It also plays a role in several non-neoplastic and neoplastic processes, especially in the enhancement of proliferation, migration and invasion; by triggering cell–tumour microenvironment interaction as well as angiogenic processes [28]. CXCR4 is known to be overexpressed in multiple solid (such as breast, ovarian and prostate cancer as well as melanoma) as well as hematologic cancer types [26,27]. The expression of this receptor in cancer cells has also been linked to metastasis to tissues with high C-X-C motif chemokine 12 (CXCL12)-concentration, e.g., lungs, liver and bone marrow [26,27]. 68Ga-Pentixafor as radio ligand for CXCR4 has been proven an alternative to 18F- fluorodeoxyglucose (FDG) PET, showing clearly higher detection rates and a better tumour-to-background contrast [29]. It also has to be discussed as a potential candidate for directed endo-radiotherapy [30].

As new therapy approaches are needed in order to battle the incidence peak of MPM expected within the upcoming decade, we aim to analyse histological MPM specimens for their MSLN and CXCR4 protein expression levels. As strong immunohistochemically staining has been proven to be associated with radio ligand accumulation in both cases [21,22,29], a significant portion of high expressing tumours within the MPM may open the field for this sophisticated treatment approach in the near future. Especially for patients showing resistance to current therapies such as platin-based agents, this could be a promising alternative to enhance patients’ outcomes.

## 2. Results

### 2.1. Clinicopathological Data of Patients

Our clinical cohort encompasses 105 patients. Overall, 84 patients were males (80%) and 21 were females (20%) with a mean age date of diagnosis of 65 years (median: 65, range: 34–82). With 102 patients, survival data were available and served for statistical analyses. At the time of data collection, 88 patients had succumbed to death, while 14 patients were alive. No data of follow-up were available for three patients. Analysis of the survival data revealed a median survival of 18.6 months. Without censoring of patients, mean survival was 23.4 months (95% confidence interval of 9.6–30.7 months, minimum: 1.2 months, maximum: 91.3 months). Analysis also revealed a median progression-free survival (PFS) of 7.5 months. Without censored patients, the mean was 12.2 months (95% confidence interval of 5.9–12.3 months, minimum: 1.9 months, maximum: 64.7 months). Regarding the morphology of MPM, we identified 96 patients (91%) with epithelioid subtype. Five patients showed a biphasic subtype (5%) and in four patients the sarcomatoid subtype of MPM (4%) was identified. Pleural decortication was performed in 70% of the patients, while pleurodesis was performed in 20% of patients. Five percent of patients underwent pleuropneumectomy. For another five percent of patients, we could not assign the status of surgical therapy due to anonymization of the data. All patients underwent varying cycles of platinum-based therapy with either cis- or carboplatin. Overall, 42 of the patients presented with stable disease (SD) (40%), seven patients showed a partial response (PR) (7%), and 54 patients presented with a progressive disease (PD) (51%). The radiological response could not be assessed for two of the patients (2%). Basic statistical results of survival analysis and association to clinicopathological data are shown in  Supplementary Table S1.

### 2.2. CXCR4 Expression in Human MPM Is Associated with Clinical Factors

**Figure 1:**
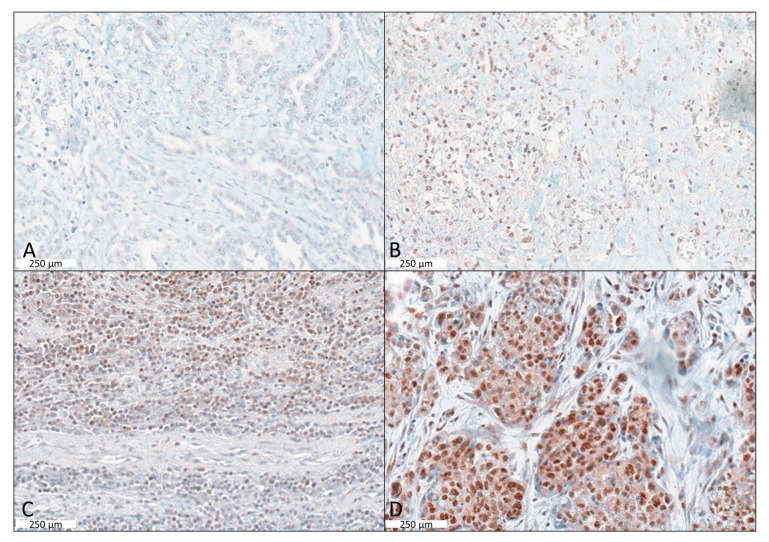
Cases of immunohistochemically CXCR4 staining representing the scoring 0–3. (**A**) IHC negative epithelioid MPM (Score 0). (**B**) Immunohistochemically weak nuclear positive MPM (Score 1). (**C**) Immunohistochemically moderate to strong nuclear positive MPM (Score 2). (**D**) Immunohistochemically strong nuclear expressing MPM (Score 3). Scale bar indicates 250 µm.

Overall, 81 tumours (77.1%) showed immunohistochemical CXCR4 protein expression (Figure 1) with 30 (28.6%) with Score 1; 24 (22.9%) with Score 2; and 27 (25.7%) with Score 3, respectively. Taken together, 48.6% of all samples showed an overall strong staining with a score of 2 or higher. Between CXCR4 expression and tumour characteristics, including tumour progression (*p* = 0.326) under platinum chemotherapy, we did not find significant associations. However, a remarkably strong positive staining of CXCR4 (Score 3) was observed only in progressive cases, with only a few (7.4%, n = 4/54) staining negative samples in this group.

### 2.3. MSLN Expression in Human MPM Is Associated with Clinical Factors

**Figure 2:**
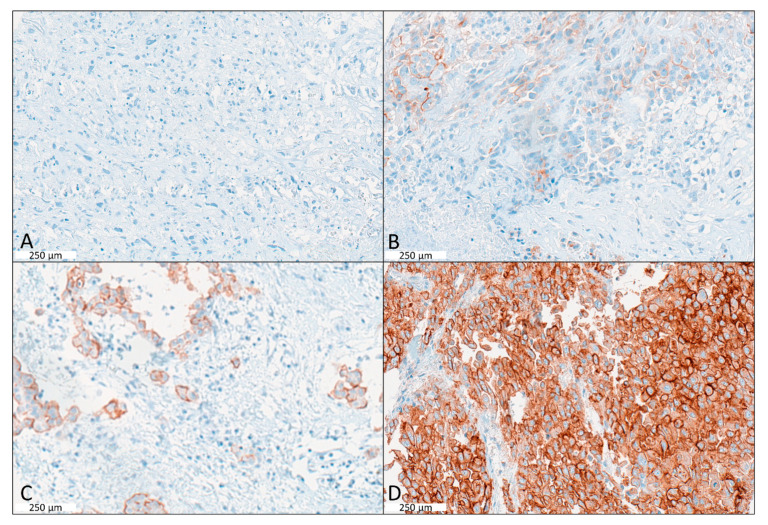
Examples of Immunohistochemically MSLN staining scores. (**A**) IHC of sarcomatoid MPM being negative for MSLN (Score 0). (**B**) IHC of low cytoplasmic expression in epithelioid MPM with score 1. (**C**) IHC showing moderate cytoplasmic expression of MSLN (Score 2). (**D**) Immunohistochemically strong cytoplasmic expressing MPM with a score of 3. Scale bar indicates 250 µm.

**Figure 3:**
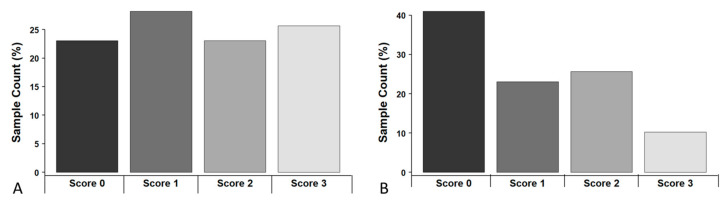
Distribution of IHC expression, based on the semi-quantitative scoring system; (**A**) CXCR4, and (**B**) MSLN.

Overall, 62 tumours (59.0%) showed MSLN protein immunohistochemical expression (Figure 2.) with 24 (22.9%) with Score 1; 27 (25.7%) with Score 2; and 11 (10.5%) with Score 3, respectively (Figure 1B). Overall, 36.2% of all samples showed an overall strong staining with a score of 2 or above. None of the tumour characteristics (e.g. histology) were significantly associated with MSLN expression (Supplementary Table S1). A summarized plot for immunohistochemical (IHC) expression of both markers based on the scoring system is shown in Figure 3.

### 2.4. Survival Analysis

We did not find any association with patients’ gender by analysing the survival data (*p* = 0.2643). Despite of the small case numbers, biphasic and sarcomatoid subtypes were included in the analysis and showed significantly shortened overall survival (OS) of patients with *p* = 0.0036. Regarding functional variations in the different tumour biology between epithelioid and sarcomatoid/biphasic MPM, only tumours of the epithelioid histological subtype were considered for the survival analysis. A subgroup for those cases only is not reasonable due to the small subset size.

**Figure 4:**
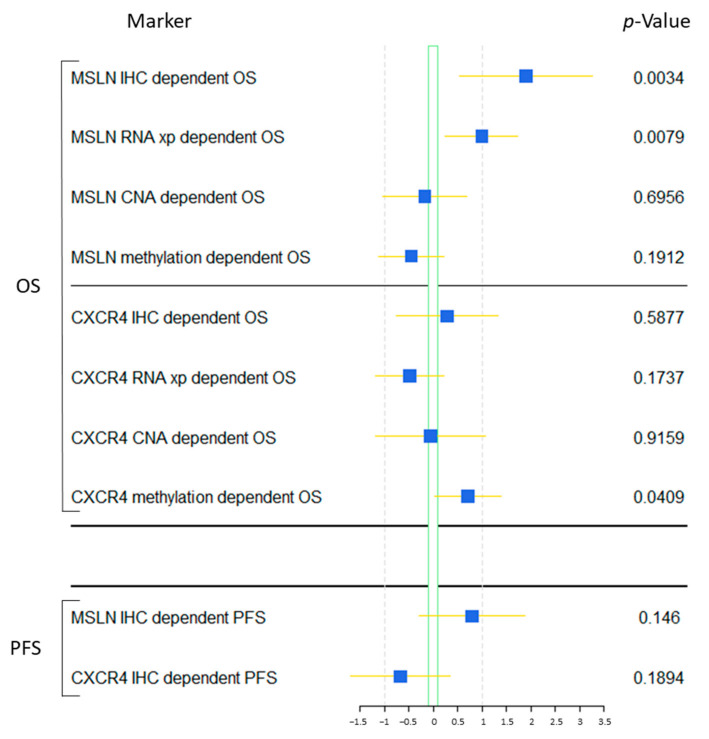
Impact of MSLN and CXCR4 expression, CNA and methylation parameters on overall and progression-free survival. This forest plot shows associations of protein expression of MSLN and CXCR4 on overall survival (OS) as well as progression-free survival (PFS) of the investigated patients. In addition, gene expression, CNA, and methylation data of CXCR4 as well as MSLN from the TCGA data base were analysed with survival data.

No influence of CXCR4 protein expression on overall survival (*p* = 0.715, hazard ratio (HR): 0.94 [95% CI: 0.67–1.31]) as well as progression-free survival (*p* = 0.494, HR: 1.11 [95% confidence interval: 0.82–1.50]) could be observed. Even when grouping in high-vs.-low expression (cut-off < score 2), no significant association could be revealed (OS: *p* = 0.588, HR (low Group): 1.22 [95% CI: 0.59–2.52]; PFS: *p* = 0.189, HR (low Group): 0.62 [95% CI: 0.31–1.37]) (Figure 4). 

In contrast, the results of the MSLN staining revealed a significant association between low protein expression and shortened overall survival (*p* = 0.012, HR: 0.62 [95% CI: 0.42–0.91]). Especially when grouped in high-vs.-low (cut-off < score 2), the group of low expressing cases showed a significant higher risk to succumb to the disease earlier (*p* = 0.003, HR: 3.74 [95% CI: 1.46–9.56]) (Figure 4). Whereas, MPM samples being negative for MSLN showed a 3-year OS rate of only 14.4% and a median survival of approximately 11.5 months, the group of MPM with strong MSLN immuno-expression shows up with a 3-years-overall-survival rate of 65.3% and a median survival of 44.8 months. However, no influence of MSLN protein expression on progression-free survival under platinum-based therapy could be observed (continual values: *p* = 0.206, HR: 0.80 [95% CI: 0.58–1.13]; grouped: *p* = 0.146, HR (low Group): 1.74 [95% CI: 0.82–3.68]) (Figure 4). Nevertheless, especially for the first two years, a clear separation between both groups can be seen, resulting in a 1-year-progression-free survival rate of 47.6% for the high group versus 18.6% for the low group with a median progression-free survival time of 10.1 vs. 7.3 months.

### 2.5. Validation of the Results and Biological Regulation Using the TCGA Data Set

For the *MSLN* gene expression pattern, a similar distribution as seen in the IHC can be observed. Overall, 55 samples (63.2%) show no or only minor *MSLN* expression, whereas 11.5% show outstanding mRNA levels. Contrarily, *CXCR4* gene expression differs from the results obtained by IHC, presenting 69% of samples without noteworthy gene expression. Only 3 samples (3.4%) show outstanding mRNA levels.

Cox-regression (COXPH) revealed similar results regarding overall survival in dependence of gene expression. The results of *MSLN* revealed a significant association between low gene expression and shortened overall survival (*p* = 0.008, HR: 0.64 [95% CI: 0.49–0.86]) (Figure 4A). For CXCR4, no significant influence on survival could be observed (*p* = 0.174, HR: 1.08 [95% CI: 0.87–1.35]). However, the methylation of the CXCR4 gene locus seems to slightly influence overall survival (*p* = 0.041), but does not impact patient outcomes individually (Figure 4).

Both gene expression patterns seem to be strongly dependent on gene locus methylation. Especially MSLN (*p* = 1.14 × 10^−9^, r = −0.60) seems to be strongly dependent on CpG methylation, leading to absent mRNA expression when methylated above 50%. However, CXCR4 also shows a significant correlation between those (*p* = 2.55 × 10^−8^, r = −0.55). Moreover, gene expression does not seem to be influenced by differences in CNAs, although there seems to be a light trend of higher MSLN mRNA expression when amplified and lower gene expression levels when heterozygous deleted (MSLN: *p* = 0.06478; CXCR4: *p* = 0.4461).

## 3. Discussion

The incidence of MPM is still increasing and there are only limited therapeutic options for this severe disease. Basic therapy approaches encompassing surgical resection are considered as therapy options in early stages. Radiation therapy has only palliative benefits for patients [3,7]. Several therapeutic agents have been tested over the past years. Experiences with inhibitors of the epidermal growth factor (EGFR)- and vascular endothelial growth factor (VEGF)-pathway have been proven to be disappointing [3]. This has brought up novel trials such as, e.g., ranpirnase, steroid receptor coactivator (src) kinase, mesothelin and vaccine approaches [3]. This unsatisfying situation makes it obvious that novel treatment strategies are urgently required.

As shown in the mouse model by Hatterer et al., a potential therapeutic assay could be possible by using monoclonal antibodies (mAb) addressing different membrane MSLN-epitopes spanning the more proximal membrane-regions, and there showing a more effective antibody-dependent cell mediated cytotoxicity [20]. According to this data, from the pathologic view, it might make sense to define MPM-patient groups with higher MSLN-expression levels as potential responders. More than half (59%) of the investigated MPM samples showed strong protein expressions of MSLN (10.5% of them at high expression (Score 3)). Still, more than one-third (36.2%) of all samples showed an overall strong staining (Score ≥ 2). A congruent *MSLN* gene expression pattern, similar to the IHC distribution, was observed within our collective. Even when the outstanding high expressing cases are still a minority, after upfront immunohistological patient stratification, ImmunoPET with anti-mesothelin antibodies and subsequent anti-mesothelin antibody-drug conjugate treatment is still a promising alternative, especially for these certain cases [21].

It is noteworthy that a strong positive staining reaction against CXCR4 has been observed only in progressive cases. Overall, more than 3/4 (77.1%) of the analysed tumours showed CXCR4 protein expression, with nearly half (48.6%) of all samples showing a strong IHC staining with a score of 2 or above and one-fourth (25.7%) of them at high expression levels (Score 3). These overall high expression levels are making MPM especially interesting for Pentixafor-bound radioisotope treatment [29]. It has to be discussed how far those patients might be discriminated for potential CXCR4-based targeted endo-radiotherapy [30]. 

In general, besides essential biological differences between the different histological subtypes, there are limitations in studying MPM based in the recordings: classification is surgically based only, leading to severe problems in determining non-resected patients [7]. Additionally, within our collective, classifying those cases was based on surgical and TNM-staging. Moreover, as shown in the literature, clinical trials considering MPM-therapy are often rather small, due to the rareness of this disease, and thus hard to compare [7]. Systemic and combined chemotherapy is the only therapeutic option in the majority of cases with advanced diseases showing very limited response rates with median survival of <1 years [3,7]. In our study, the outcome has to be described accordingly.

Furthermore, the gene expression pattern of MSLN seems to be strongly dependent on gene locus methylation, which may be a reason why potentiating effects have been observed in experimental ADCC-assays only by addressing specific membrane regions [20]. Corresponding to *MSLN*-gene expression, *CXCR4*-expression patterns also seem to be strongly depending on gene locus methylation, but do not seem to be influenced by differences in copy number alterations (CNAs). There seems to be a light trend of higher MSLN gene expression if amplification is observed and lower gene expression levels when heterozygous deletion is observed.

## 4. Materials and Methods

### 4.1. The Demographic Data

Formalin-fixed paraffin-embedded (FFPE) tumour samples from a total of 105 MPM patients have been analysed. For the classification of tumours, the respective literature of the WHO classification guidelines has been used (2004) [31],.TNM-staging is based on the Union internationale contre le cancer (UICC) guidelines of classification [32] and was confirmed by two pathological experts (JW, TM). MPM patients were treated either at the West German Cancer Centre or at the West German Lung Centre (Essen) (between 2006 and 2009) or the Helios Klinikum Emil von Behring (Berlin) (between 2002 and 2009). Inclusion criteria for this study were the availability of sufficient tumour material as well as a complete set of follow-up and treatment data. We summarized the demographic data in Table 1.

### 4.2. Clinicopathological Data

The majority of the investigated tumours presented with epithelioid morphology, while only five cases were classified as biphasic and four cases as sarcomatoid. All samples were collected prior to platinum-based treatment. Termination of study surveillance was on 31 August 2014.

The modified Response Evaluation Criteria in Solid Tumours (modRECIST), which is used to assess radiological response in MPM, served for the evaluation of response data of the cohort [33]. Remission was defined by partial response (PR) and complete response (CR) or by progressive disease (PD) and stable disease (SD) [34].

Furthermore, gene expression data (whole-transcriptome sequencing) as well as copy number alterations and methylation data (bisulphite whole-methylome sequencing) of 87 malignant pleural mesothelioma patients were retrieved from “The Cancer Genome Atlas (TCGA)” database (consideration of National Cancer Institute, National Human Genome Research institute, Bethesda, MD, USA).

The Ethics Committee of the Medical Faculty of the University Duisburg-Essen approved the study (identifier: 14-5775-BO), which was performed retrospectively. The investigation is in line with the principles of the Helsinki Declaration.

### 4.3. Immunohistochemistry

FFPE samples were cut to slides of 4μm thickness and used for hematoxylin and eosine (H&E) staining as well as for immunohistochemistry (IHC). Tissue microarrays (TMA) were created from paraffin blocks for systematic immunohistochemical investigations. Three plugs with a diameter of 0.6 mm were transferred from various areas of each tumour block to consider the potent heterogeneity of the tumours. If available, plugs comprising healthy lung tissue and modest pleura were taken from each sample, serving as negative control. Immunohistochemical staining was performed according to standard protocols using an automated staining device (Ventana BenchmarkUltra, Roche Pharma AG, Grenzach-Wyhlen, Germany). After validation on normal pleura samples, the immunohistochemical staining was carried out with a primary monoclonal antibody (clone 12g5; Thermo Fisher Scientific Inc., Waltham, MA, USA) directed against CXCR4. Dilution was set with 1:50. Samples were pre-treated for antigen retrieval by heating in citrate buffer at 90 °C for 32 min at pH 6 (Ultra Cell Conditioning Solution II, Ventana Medical Systems, Basel, Switzerland). For MSLN staining, a primary monoclonal antibody (clone 5B2, BioGenex Life sciences Pvt. Ltd., Fremont, CA, USA) was used. Dilution was set at 1:100. Additionally, samples were pre-treated for antigen retrieval by heating in citrate buffer for 24 min at 95 °C at pH 6 (Ultra Cell Conditioning Solution II, Ventana Medical Systems, Basel, Switzerland).

A consultation light microscope (Nikon Eclipse 80i, Nikon Ltd., Dusseldorf, Germany) was used to evaluate immunohistochemical protein expression of CXCR4- and MSLN. The evaluation based on a semi-quantitative IHC scoring system. This system assesses the percentage of tumour cells with positive staining irrespective of intensity of staining. Score 0 means there was no immunohistochemical signal; Score 1 means approximately 1–10% positive tumour cells and means weak expression. Score 2 shows moderate with 11–50% tumour cells being positive, while a score of 3 means strong expression with >50% of positive tumour cells [35,36].

### 4.4. Statistical Analysis

Statistical and graphical analyses were performed with the R statistical programming environment (v3.2.3). Before starting the explorative data analysis, the Shapiro–Wilk-test was applied to test for the normal distribution of the data. For dichotomous variables, either the Wilcoxon Mann–Whitney rank sum test (non-parametric) or two-sided student’s *t*-test (parametric) was applied. For ordinal variables with more than two groups, either the Kruskal–Wallis test (non-parametric) or ANOVA (parametric) was used to detect group differences. Double dichotomous contingency tables were analysed using Fisher’s exact test. To test the dependency of ranked parameters with more than two groups, the Pearson’s Chi-squared test was used. Correlations between metric variables were tested by using the Spearman’s rank correlation test as well as the Pearson’s product moment correlation coefficient for linear modelling.

Analysis of OS was performed by producing single-factorial and combined Kaplan–Meier plots. Survival analyses were performed by the Cox-regression model (COXPH-model). Statistical significance has been determined by using the likelihood ratio test, Score (logrank) test and the Wald test. All *p*-values were adjusted using the false discovery rate (FDR), with the aim to overcome multiple testing issues. A value of *p* < 0.05 was defined as statistically significant after adjustment.

## 5. Conclusions

MPM to date is still a challenging tumour entity with an increasing number of cases and poor outcomes. Standard therapy as well as combined chemotherapeutic regimens do not lead to ground-breaking benefits. Novel antigens are currently under experimental and clinical evaluation. As shown in the literature, MSLN and CXCR4 are potential agents for targeted anti-tumour or endo-radioligand therapy. Our results show significant tissue expression levels, for both CXCR4 and MSLN protein, in a major portion of clinical MPM samples. One-third of patients showed outstanding immunoexpression of one or the other of those markers, making them especially interesting candidates for radioligand-based PET/CT diagnostics and follow-up as well as endo-radiotherapy.

## Figures and Tables

**Table 1:** Overview of clinicopathological data.

Number of Patients	105
Gender	
male	84
female	21
unknown Gender	0
Histological subtype	
epithelioid	96
biphasic	5
sarcomatoid	4
Age	
Mean|Median age at diagnosis (years)	65|65
Range (years)	34–82
OS	
Deceased	88
Alive	14
Lost-of-FU	3
Median|Mean OS (months)	18.6|23.4
Range (months)	1.2–91.3
PFS	
Partial remission (initial)	7
Stable disease (initial)	42
Progressive disease (initial)	54
Unknown response	2
Median|Mean PFS (months)	7.5|12.2
95% CI	5.9–12.3

## Data Availability

The data presented in this study are available on request from the corresponding author. The data are not publicly available due to privacy restrictions.

## Supplementary Materials

The following supporting information can be downloaded at: https://www.mdpi.com/article/10.3390/ijms24076356/s1.
